# Inhibition of the Activin Receptor Type-2B Pathway Restores Regenerative Capacity in Satellite Cell-Depleted Skeletal Muscle

**DOI:** 10.3389/fphys.2018.00515

**Published:** 2018-05-24

**Authors:** Luigi Formicola, Alice Pannérec, Rosa Maria Correra, Barbara Gayraud-Morel, David Ollitrault, Vanessa Besson, Shahragim Tajbakhsh, Jennifer Lachey, Jasbir S. Seehra, Giovanna Marazzi, David A. Sassoon

**Affiliations:** ^1^UMR S 1166 French National Institute of Health and Medical Research, France and the Institute of Cardiometabolism and Nutrition, Stem Cells and Regenerative Medicine, University of Pierre and Marie Curie Paris VI, Paris, France; ^2^Centre National de la Recherche Scientifique URA 2578, Institut Pasteur, Stem Cells and Development, Paris, France; ^3^Acceleron Pharma, Cambridge, MA, United States; ^4^Ember Therapeutics, Watertown, MA, United States

**Keywords:** muscle atrophy, muscle regeneration, muscle stem cell niche, satellite cells, TGFβ signaling

## Abstract

Degenerative myopathies typically display a decline in satellite cells coupled with a replacement of muscle fibers by fat and fibrosis. During this pathological remodeling, satellite cells are present at lower numbers and do not display a proper regenerative function. Whether a decline in satellite cells directly contributes to disease progression or is a secondary result is unknown. In order to dissect these processes, we used a genetic model to reduce the satellite cell population by ~70–80% which leads to a nearly complete loss of regenerative potential. We observe that while no overt tissue damage is observed following satellite cell depletion, muscle fibers atrophy accompanied by changes in the stem cell niche cellular composition. Treatment of these mice with an Activin receptor type-2B (AcvR2B) pathway blocker reverses muscle fiber atrophy as expected, but also restores regenerative potential of the remaining satellite cells. These findings demonstrate that in addition to controlling fiber size, the AcvR2B pathway acts to regulate the muscle stem cell niche providing a more favorable environment for muscle regeneration.

## Introduction

Chronic degenerative muscle diseases eventually lead to a collapse in the ability of muscle to regenerate. In the case of Duchenne muscular dystrophy (DMD), afflicted boys show subtle motor defects during early postnatal life that rapidly increase with age leading to paralysis and premature death (Parker et al., 2005; Tabebordbar et al., 2013). It has been proposed that the loss of regenerative capacity in DMD results from an exhaustion of the muscle progenitor satellite cell pool during disease progression (Blau et al., 1983, 1985; Heslop et al., 2000; Jiang et al., 2014; Lu et al., 2014). During the early phase of DMD, muscle undergoes continuous rounds of degeneration/regeneration but eventually regenerative competence declines accompanied by a decrease in satellite cell number (Wallace and McNally, 2009; Mann et al., 2011; Tabebordbar et al., 2013). Following a genetically induced depletion of satellite cells, muscle damage leads to a replacement of myofibers by fibrosis and fat deposition (Lepper et al., 2011; Murphy et al., 2011; Sambasivan et al., 2011). Whether the decline in satellite cells contributes to disease progression is unclear, in part since dissecting the relative contribution of a decreased satellite cell number in mouse DMD models is hampered by the fact that these models do not reflect the severity of disease progression in boys (Hoffman et al., 1987; Coulton et al., 1988; Tabebordbar et al., 2013).

We wished to address whether a reduction in the number of satellite cells has a direct effect upon the muscle tissue in the absence of injury and in turn, whether changes in the muscle tissue have an adverse effect upon satellite cell function. This question is particularly relevant as it has been demonstrated that small numbers of satellite cells engraft with high efficiency into healthy skeletal muscle (Collins et al., 2005; Sacco et al., 2008), thus a decline but not complete elimination of the satellite cell pool should be sustainable. Furthermore, satellite cells obtained from mdx mice, a model for Duchenne, contribute robustly when engrafted into healthy muscle (Boldrin et al., 2015). These observations raise the question as to why a reduced satellite cell population cannot replace damaged muscle fibers in diseased muscle?

A number of secreted growth factors have been shown to regulate satellite cell function. Different combinations of TGFβ superfamily receptors and ligands control muscle growth, progenitor activation, fibrosis and ectopic bone and fat formation (Yamaguchi, 1995; Glass, 2010; Sako et al., 2010; Serrano et al., 2011; Bonaldo and Sandri, 2013; Sartori et al., 2013). Myostatin (MST) is a high affinity ligand for the activin receptor-2B (AcvR2B) and a potent negative regulator of muscle growth that blocks myogenic progression (McPherron et al., 1997; Thomas et al., 2000; Lee and McPherron, 2001). *Myostatin*-null mice display a pronounced increase in muscle mass coupled with reduced fibrosis following injury (McPherron and Lee, 2002; McCroskery et al., 2005). MST has been shown to stimulate fibrosis (McCroskery et al., 2005; Artaza et al., 2008; Z Hosaka et al., 2012) whereas suppression of MST, using a soluble form of the AcvR2B, has been used to treat congenital myopathies, sarcopenia and cachexia resulting in reduced fibrosis (Wagner et al., 2002; Siriett et al., 2006; Morrison et al., 2009; Cadena et al., 2010; Zhou et al., 2010; George Carlson et al., 2011; Lawlor et al., 2011; Pistilli et al., 2011; Chiu et al., 2013; Lach-Trifilieff et al., 2014). Follistatin (FST) binds to and blocks MST activity results in muscle hypertrophy (Lee and McPherron, 2001; Amthor et al., 2004; Lee et al., 2010). Other growth factors such as insulin-like growth factor 1 (IGF-1) are potent activators of muscle growth that act in part by blocking the downstream MST-induced pathway (Bodine et al., 2001; Rommel et al., 2001; Trendelenburg et al., 2009; Oberbauer, 2013). While manipulation of the AcvR2B pathway provides a basis for development of therapeutic approaches for degenerative myopathies (Glass, 2010; Zhou and Lu, 2010; Ceco and McNally, 2013; Tabebordbar et al., 2013), it remains unaddressed whether these therapies act via a primary effect upon myofibers or target additional cell types including satellite cells and/or the stem cell niche. Satellite cells are highly responsive to neighboring cell populations *in vivo* (Ten Broek et al., 2010; Yin et al., 2013) including myofibers, vessels, pericytes, and fibroadipogenic progenitors (FAPs) (Joe et al., 2010; Uezumi et al., 2010; Dellavalle et al., 2011; Pannérec et al., 2012, 2013) as well as invading macrophages following injury (Kharraz et al., 2013; Tabebordbar et al., 2013; Yin et al., 2013; Farup et al., 2015). While FAPs generate fat in diseased muscle, a depletion of interstitial cells including FAPs results in poor regeneration (Murphy et al., 2011), indicating that interstitial cells play a critical role in proper muscle regeneration (Murphy et al., 2011).

In this study, we used a genetic mouse model for depleting satellite cells that relies upon diphtheria toxin targeted cell death to satellite cells (Sambasivan et al., 2011). In this model, injured muscle undergoes pronounced fibrosis and fat formation coupled with a near complete loss of fiber regeneration (Lepper et al., 2011; Murphy et al., 2011; Sambasivan et al., 2011). We found that diphtheria toxin injection resulted consistently in ~70–80% satellite cell reduction, thereby providing a baseline by which we could directly assess the effects of satellite cell reduction in the absence of injury and/or underlying disease state. In addition, this model allows for a reduction of satellite cells in a single muscle group as opposed muscles that provide essential functions such as the diaphragm. We found that satellite cell depletion resulted in muscle fiber atrophy in the absence of injury. Whereas inhibition of the AcvR2B results in muscle fiber hypertrophy in muscle containing an intact satellite cell population (Morrison et al., 2009; Pistilli et al., 2011), we observed that satellite cell depleted muscles undergo a limited hypertrophic response in which muscle fiber size is only restored to levels found in intact untreated muscles. In addition to changes in the muscle fibers, we observed that PW1+ interstitial cells undergo a marked increase in cell number following AcvR2B pathway inhibition suggesting that the stem cell niche is responsive. This prompted us to test whether this change in the stem cell niche would have an impact upon the regenerative potential in muscle containing fewer satellite cells. We report here that treatment of satellite cell depleted muscle with an AcvR2B inhibitor prior to injury rescues regenerative potential concomitant with a near complete inhibition of ectopic fat formation and fibrosis. Using genetic lineage tracing, we found that the regenerated fibers originated from the residual pool of satellite cells in the satellite cell-depleted mice. These data reveal that a decline in satellite cells has a profound effect on the tissue environment compromising myogenic competence and that inhibition of the AcvR2B pathway restores the regenerative potential of a reduced satellite cell population.

## Materials and methods

### Mice models

Animal models used were C57Bl6J mice (Elevage Janvier); *PW1*^*IRESnLacZ*^ transgenic reporter mice (PW1^nlacZ^) (Besson et al., 2011); knock-in heterozygous *Pax7*^*DTR*/+^ mice, in which the diphtheria toxin receptor (DTR) is expressed under the control of the *Pax7* promoter (Sambasivan et al., 2011); *Tg:Pax7CreER*^*T*2^ mice, carrying a BAC where a tamoxifen-inducible Cre recombinase/estrogen receptor fusion protein, CreER^T2^ (Metzger and Chambon, 2001), is expressed under the control of *Pax7* (Mourikis et al., 2012); ROSA26^mTomato/mGFP^ (ROSA^mTmG^) mice (Muzumdar et al., 2007) (Jackson Laboratories). *Pax7*^*DTR*/+^ mice were crossed with *Tg:Pax7CreER*^*T*2^ and ROSA^mTmG^ to obtain Pax7^DTR/+^:Pax7CreER^T2^:ROSA^mTmG^ animals.

### Ethics statement

Approval for the animal (mouse) work performed in this study was obtained through review by the French Ministry of Education, Agreement #A751320.

### RAP-031 treatment

Five-weeks old C57Bl6 or PW1^nLacZ^ males and 10-weeks old Pax7^DTR/+^ or Pax7^DTR/+^:Pax7CreER^T2^:ROSA^mTmG^ mice were injected intra-peritoneally with RAP-031 or vehicle (TBS) 10 mg per kg^−1^.

### Tamoxifen treatment

Six weeks-old Pax7^DTR/+^:Pax7CreER^T2^:ROSA^mTmG^ mice were injected intra-peritoneally every day for 4 days with tamoxifen (250–300 ml, 20 mg/ml; Sigma Aldrich) diluted in sunflower seed oil/5% ethanol.

### Toxins injections

Mice were anesthetized by intra-peritoneal injection of ketamine (100 mg.kg^−1^) and xylazine (10 mg.kg^−1^) in sterile saline solution. A total volume of 15–30 μl was used for one single intramuscular injection of diphtheria toxin (DT) from *Corynebacterium diphtheria* (Sigma Aldrich) at 1 ng.g^−1^ of body weight or PBS into the TA muscle using 30 G Hamilton syringe. Muscle injury was induced by intramuscular injection of 40 μl of cardiotoxin from *Naja mossambica* (Latoxan) at a concentration of 10 μM.

### Western blot analysis

TA muscles were homogenized in a lysis buffer (150 mM NaCl, 50 mM Hepes pH7.6, 1% NP-40, 0.5% Sodium deoxycholate, 5 mM EDTA) supplemented with 1 mM PMSF, protease inhibitor cocktail (Roche, Cat. No. 04693124001), 20 mM NaF, 10 mM b-glycerophosphate, 5 mM Na-pyrophosphate, and 1 mM Na-orthovanadate. Equal amount of protein were separated by electrophoresis (Novex NuPAGE Bis-Tris protein gel 12%) and transferred to a PVDF membrane in 20% methanol transfer buffer. Membranes were probed with p-SMAD2 (S465/467)/ Smad3 (S423/425), SMAD2/3 total (Cell signaling) and GAPDH (Abcam). Anti-body binding was visualized using horse-radish peroxidase (HRP)-conjugated species-specific secondary antibodies (Jackson ImmunoResearch) followed by enhanced chemiluminescence (Pierce).

### FACS analysis

For fluorescence activated cell sorting (FACS), hindlimb muscles from 7 weeks old PW1^nLacZ^ mice were digested (Mitchell et al., 2010) and FACS sorted as described previously (Pannérec et al., 2013): briefly, muscles were minced and incubated with a solution of collagenase/dispase for 90 min at 37°C, then cells were washed three times in BSA 0.2% (Jackson Immunoresearch) diluted in Hank's Balanced Salt Solution (HBSS) (Life Technologies) and then incubated for 1 h on ice with the following primary antibodies at a concentration of 10 ng.ml^−1^: rat anti-mouse CD45-APC (BD Biosciences), rat anti-mouse Ter119-APC (BD Biosciences), rat anti-mouse CD34-E450 (eBiosciences), rat anti-mouse Sca1-A700 (eBiosciences), and rat anti-mouse PDGFRα-PE (CD104a, eBiosciences). Cells were washed and incubated with C_12_FDG, 60 μM, (Life Technologies) 1 h at 37°C and analyzed and sorted on a FACSAria (Becton Dickinson) with appropriate isotype matching controls as previously reported (Pannérec et al., 2013). Ter119^pos^ and CD45^pos^ cells were negatively selected and the remaining cells were gated based on the other markers: satellite cells were sorted in the CD34^pos^Sca1^neg^ fraction, while PDGFRα^pos^ and PDGFRα^neg^ PICs were sorted in the CD34^pos^Sca1^pos^C_12_FDG^pos^(PW1^pos^) fraction (Pannérec et al., 2013). At least 3 independent experiments were performed for CTL and RAP-031-injected mice groups.

### Cell culture

For transwell co-culture experiments, FACS-sorted cell populations were maintained in high serum medium (GM) as described previously (Pannérec et al., 2013). Satellite cells were plated in the lower chamber at a density of 100 cells/cm^2^. PICs were plated on the membrane of the insert well (1 μm pore size, BD Biosciences) at 3,000 cells per cm^2^. Cells were grown for 1 day in GM before adding the following factors: recombinant myostatin (R&D Systems) was added at 0, 20, 200, or 2,000 ng.mL^−1^; human follistatin blocking antibody (αFST) (R&D Systems), mouse IGF-1 blocking antibody (αIGF1) (R&D Systems) and isotype matched IgG (R&D Systems) were used at 4 μg.mL^−1^ (R&D Systems). After 2 days, cells were fixed with 4% (w/v) paraformaldehyde (Sigma Aldrich) and the number of cells per colony was counted. At least 3 independent experiments were performed for each condition.

### Gene expression analysis

RNA extracts were prepared from freshly FACS sorted cells using RNeasy micro-kit (Qiagen). cDNA was generated by random-primed reverse transcription using the SuperScript First Strand kit (Life Technologies). cDNAs were analyzed by semi-quantitative PCR using the ReddyMix PCR Master Mix (Thermo Scientific) under the following cycling conditions: 94°C for 5 min followed by 32 cycles of amplification (94°C for 30 s, 60°C for 30 s and 72°C for 1 min) and a final incubation at 72°C for 10 min. Primers are listed in Supplementary Table 1.

### Histological analyses

*Tibialis anterior* (TA), *soleus, plantaris*, and *gastrocnemius* muscles were removed, weighed and snap frozen in liquid nitrogen-cooled isopentane (Sigma Aldrich) as previously described (Mitchell et al., 2010; Pannérec et al., 2013). In the case of Pax7^DTR/+^:Pax7CreER^T2^:ROSA^mTmG^ mice, TA muscles were fixed 2 h in 4% (w/v) paraformaldehyde (Sigma Aldrich), incubated overnight in 20% (w/v) sucrose (Sigma Aldrich) and frozen in liquid nitrogen-cooled isopentane (Sigma Aldrich). Muscles were cryosectioned (7 μm) before processing. Cryosections were stained with haematoxilin and eosin (H&E) (Sigma Aldrich). Fat tissue was detected by Oil-Red O staining (Sigma Aldrich): cryosections were fixed in 10% formalin (Sigma Aldrich) for 5 min at 4°C, rinsed in water and then 100% propylene glycol (Sigma Aldrich) for 10 min, stained with Oil red O (Sigma Aldrich) for 10 min at 60°C, placed in 85% propylene glycol for 2 min and rinsed in water. Nuclei were counterstained with Mayer's Hematoxylin Solution (Sigma). Collagen deposition was detected by Sirius Red staining (Sigma Aldrich): cryosections were stained with Mayer's haematoxylin (Sigma Aldrich) for 10 min, rinsed in running tap water for 20 min, then stained with 1.3% (w/v) Picro-sirius Red solution (Sigma Aldrich) for 45 min and washed twice in acidified water. The fat and fibrotic index was calculated as percent area of fat (red-colored Oil-Red O-stained areas) and collagen (pink-colored areas) of the total tissue area using NIH Image J Software (http://rsbweb.nih.gov/ij/) as reported previously (Tanano et al., 2003; Mozzetta et al., 2013).

For immunofluorescence, TA cryosections were fixed in 4% (w/v) paraformaldehyde and processed for immunostaining as described previously (Mitchell et al., 2010; Pannérec et al., 2013). Primary antibodies were: PW1 (Relaix et al., 1996) (rabbit, 1:3,000), Pax7 (mouse, Developmental Studies Hybridoma Bank, 1:15), M-Cadherin (mouse, Nanotools, 1:100), Ki67 (mouse, BD Biosciences, 1:100), Ki67 (rabbit, Abcam, 1:100, GFP (chicken, Abcam, 1:500), laminin (rabbit, Sigma, 1:100). Antibody binding was revealed using species-specific secondary antibodies coupled to Alexa Fluor 488 (Molecular Probes), Cy3 or Cy5 (Jackson Immunoresearch). Nuclei were counterstained with DAPI (Sigma Aldrich). For quantitative analyses of immunostained tissues, positive cells for at least 300 myofibers were counted in at least 5 randomly chosen fields per muscle section, *n* = 3 animals for each group. Fiber size distribution was measured from cryosections obtained from the mid-belly of TA stained with laminin, images were captured on a Zeiss AxioImagerZ1 microscope, and morphometric analysis was performed using MetaMorph7.5 (Molecular Devices). For uninjured muscles, at least 400 fibers from randomly chosen fields were analyzed per muscle section, *n* = 3 animals for each group. For injured muscles, between 200 and 575 fibers from randomly chosen fields were analyzed per muscle section, *n* = 3 animals for each group.

### Statistical analysis

All statistics were performed with an unpaired Student's *t*-test using the GraphPad software. Values represent the mean ± s.e.m. ^*^p < 0.05 ^**^p < 0.01 ^***^p < 0.001 and ^****^p < 0.0001.

## Results

### Satellite cell depletion results in muscle fiber atrophy and reduces hypertrophy in response to AcvR2B pathway inhibition

**Figure 1:**
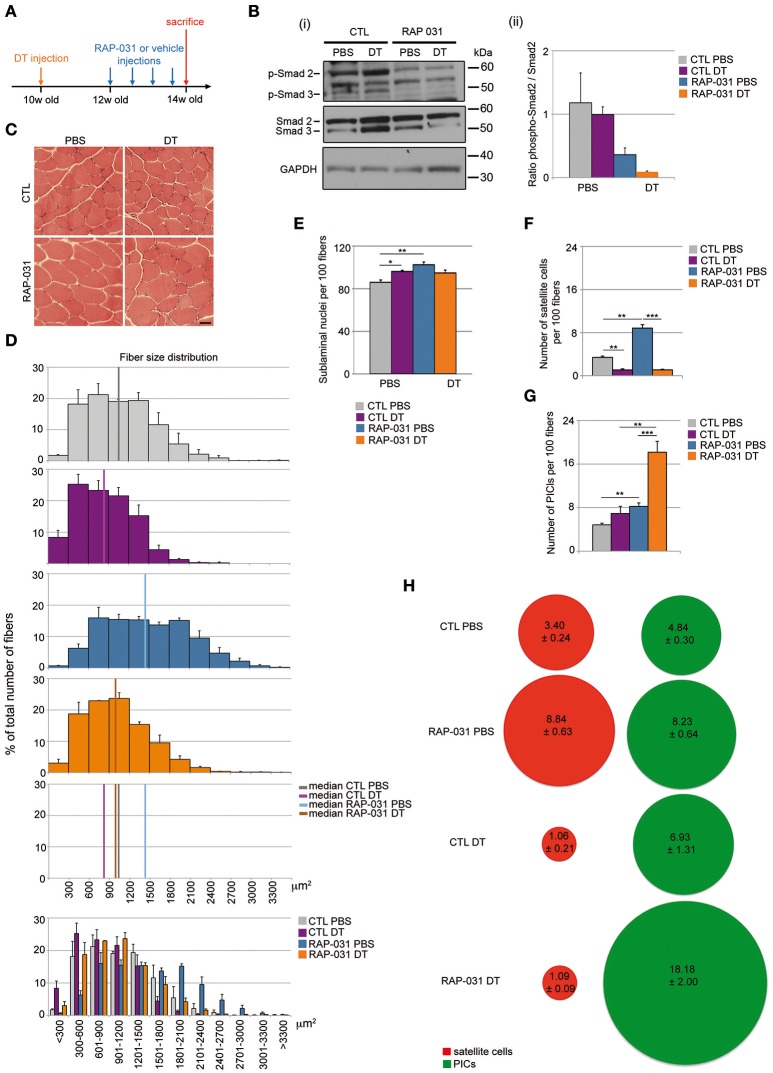
RAP-031 treatment rescues the reduction of muscle fiber size induced by satellite cell depletion and increases PICs in satellite cell-depleted muscle. **(A)** Strategy: the right TA of 10 week-old *Pax7*^*DTR*/+^ males was injected with diphteria toxin (DT) to deplete satellite cells while the contralateral muscle was injected with PBS 2 weeks before RAP-031 treatment. Mice were intraperitoneally injected with RAP-031 or vehicle (CTL) twice a week for 2 weeks and sacrificed 14 days after the first injection. **(B)** Western Blot of TA protein extracts with antibodies against phospho-Smad2/3, total Smad2/3 and GAPDH. **(i)** Representative image of the blots. Molecular size in kDa is shown on the right side. **(ii)** Ratio between levels of phospho-Smad2 and levels of total Smad2. The graph shows a marked reduction in the ratio pSmad2/Smad2 in RAP-031 muscles, indicating an inhibition of the AcvR2B pathway following systemic RAP-031 treatment. *N* = 2 muscles per group. **(C)** Cross-sections images of TA muscles from CTL (Upper) or RAP-031 (Lower) mice injected with PBS (Left) or DT (Right) stained with hematoxylin and eosin. Scale bar, 60 μm. **(D)** Fiber size distribution in CTL PBS (gray), CTL DT (purple), RAP-031 PBS (blue), and RAP-031 DT (orange) TAs. RAP-031 treatment rescued the reduction of muscle fiber size induced by satellite cell depletion. Values represent the mean number ± s.e.m. per 100 fibers for each size. *N* = 3 animals for each group. Median of fiber area distributions are shown in the graphs. **(E)** Quantification of the number of sublaminal nuclei per 100 fibers in TA from CTL and RAP-031 mice injected with PBS or DT. RAP-031 treatment did not result in myonuclei addition when satellite cells are depleted. Values represent the mean number ± s.e.m. per 100 fibers. At least 300 fibers from randomly chosen fields were counted for each animal, *n* = 3 animals were considered for each group. **(F,G)** Quantification of satellite cells **(E)** and PICs **(F)** per 100 fibers in TA from CTL and RAP-031 mice injected with PBS or DT. **(H)** Ratio between PICs (green) and satellite cells (red) per 100 fibers in TA from CTL and RAP-031 mice injected with PBS or DT was profoundly altered in DT-injected mice after RAP-031 treatment. In **(E–G)**, satellite cells were determined as M-Cadherin^pos^ cells underneath the basal lamina, PICs were determined as interstitial PW1^pos^ M-Cadherin^neg^ cells. In **(E–G)**, values represent the mean number of positive cells ± s.e.m. per 100 fibers. At least 300 fibers from randomly chosen fields were counted for each animal, *n* = 3 animals were considered for each group. For all values, ^*^p < 0.05, ^**^p < 0.01, and ^***^p < 0.001.

We used the *Pax7*^*DTR*/+^ mouse model in which the DT receptor is expressed under the control of *Pax7* such that injection of DT results in a substantial depletion of the satellite cell population (Sambasivan et al., 2011). Ten week-old *Pax7*^*DTR*/+^ mice were injected with DT in the *Tibialis Anterior* (TA) (CTL DT mice) and PBS in the contralateral muscle as a control (CTL PBS mice) and allowed to recover for 4 weeks (Figure 1A). We observed that DT-induced satellite cell depletion resulted in a marked reduction (20%) in fiber size indicating that satellite cells are required to maintain proper fiber size in resting muscle (median value of fiber area distribution: CTL PBS = 1058.67 ± 141.49 μm^2^; CTL DT = 831.33 ± 85.38 μm^2^) (Figures 1C,D). It has been shown previously that a soluble form of the AcvR2B (RAP-031) containing the ligand-binding site inhibits AcvR2B signaling by functioning as a decoy receptor *in vivo* and that this inhibition results in marked hypertrophy due to inhibition of MST and activin activity (Akpan et al., 2009; Koncarevic et al., 2010; George Carlson et al., 2011; Pistilli et al., 2011). We therefore tested RAP-031 in muscle containing an intact population of satellite cells (PBS-injected control referred to here as “intact”) and DT-induced satellite cell depleted mice. Mice were injected intraperitoneally with RAP-031 or vehicle (CTL) twice a week for 2 weeks (Figure 1A). In order to confirm AcvR2B inhibition following RAP-031 administration, we performed Western Blot analyses on TA muscle extracts, which revealed a 50% reduction of the ratio between the phosphorylated form of Smad2 (pSmad2) and the total amount of Smad2 in RAP-031 treated animals, regardless of the status of the satellite cell pool (both in PBS and DT muscles), thus confirming inhibition of the AcvR2B signaling following RAP-031 administration (Figure 1B). Moreover, as reported by others (Cadena et al., 2010; George Carlson et al., 2011; Pistilli et al., 2011), intact muscle exposed to RAP-031 displayed a pronounced increase in fiber size (median value of fiber area distribution: CTL PBS = 1058.67 ± 141.49 μm^2^; RAP PBS = 1435.00 ± 105.67 μm^2^) (Figures 1C,D). However, in satellite cell-depleted muscle, RAP-031 treatment rescued the reduction of muscle fiber size previously induced by DT injection, but failed to generate a net fiber hypertrophic effect (median value of fiber area distribution: RAP DT = 969.00 ± 61.25 μm^2^) (Figures 1C,D). Taken together, these data indicate that a reduced satellite cell compartment limits muscle fiber hypertrophy in response to AcvR2B inhibition.

### Depletion of the satellite cell compartment alters the stem cell niche

Fiber hypertrophy is typically accompanied by an increase in myonuclear content in intact muscle, a process referred to as nuclear accretion (Smith et al., 2001; Pallafacchina et al., 2013). We observed that RAP-031 treatment of intact muscles resulted in a marked increase in sublaminal nuclear content as compared to vehicle-injected mice (Figure 1E) consistent with previous studies (Smith et al., 2001; McCroskery et al., 2003; Zhou et al., 2010; Wang and McPherron, 2012). In contrast, we did not observe a change in sublaminal nuclei content in DT RAP-031 mice as compared to controls (Figure 1E) indicating that in presence of a reduced satellite cell pool, other progenitors with myogenic capacities (pericytes, myoPICs) are not recruited into the myofibers. We do note a minor increase in sublaminal nuclei following satellite cell depletion in the absence of RAP-031 exposure (Figure 1E), however this is likely due to a reduced fiber volume, nonetheless, we cannot rule out that a portion of the surviving satellite cells or other resident progenitor cells fuse with the muscle fibers in response to satellite cell depletion or DT-treatment.

We next sought to determine the status of satellite cells and other resident progenitor cell populations in response to satellite cell depletion in the presence or absence of RAP-031 exposure. Muscle tissue sections from PBS or DT-injected muscles from vehicle and RAP-031 treated mice were stained for PW1/Peg3 (to label PICs) and M-Cadherin (to label satellite cells) (Supplementary Figure 1). As expected (Sambasivan et al., 2011), satellite cell numbers were markedly decreased following DT injection to levels of ~25–30% as compared to levels in intact muscles (Figure 1F). Furthermore, whereas RAP-031 treatment induced a 2-fold increase in satellite cell number in intact muscles (Figure 1F), we did not observe an increase in satellite cell number in satellite cell depleted muscles in response to RAP-031 treatment (Figure 1F). These results suggest that once the satellite cell number is below a specific or threshold level, their response to AcvR2B inhibition is abrogated. As shown previously (Sambasivan et al., 2011) and observed in this study, PICs are clearly detectable following satellite cell depletion and we observed a modest increase in PICs number following satellite cell depletion as compared to intact muscles (Figure 1G). Furthermore, we observed that PICs undergo a 2-fold increase in intact muscles and a 4-fold increase in satellite cell depleted muscles following RAP-031 treatment (Figure 1G), indicating that PICs increase in number in response to AcvR2B inhibition in all conditions and that this response is increased when satellite cell numbers are reduced. Whether the increase in PICs is due to a proliferation of previously existing PICs or *de novo* PW1 expression in a subset of interstitial cells is not known and requires the generation of novel transgenic mouse models to specifically track PICs. Since we observed a deregulation of the number and proportion of muscle cell populations in our experimental conditions, we wished to better characterize the status of the muscle stem cell niche. We therefore checked for the expression of a marker of myogenic cell activation (MyoD) and a marker of cell proliferation (Ki67) in combination with markers for satellite cells (PW1, Pax7) and PICs (PW1) via immunostaining in TA cryosections. We did not detect MyoD expression in TA from any of the conditions tested (data not shown), indicating that neither DT-induced satellite cell pool depletion nor AcvR2B inhibition induced myogenic activation of satellite cells or any other muscle progenitors. In addition, we detected only rare Ki67^pos^ cells in all the conditions examined, among which few Ki67^pos^ PICs and no Ki67^pos^ satellite cell in DT RAP-031 mice (data not shown), indicating that satellite cells and a majority of PICs were in quiescent cell cycle state (G0 resting phase) at the end of the observation period, both in the PBS and DT-injected mice, and regardless of AcvR2B inhibition treatment.

We had shown previously that PICs and satellite cells are present in a ~1:1 ratio in resting muscle throughout postnatal life, whereas the constitutive loss of *Pax7*, which leads to a concomitant decrease of satellite cells and an increase in PICs such that the ratio of PICs and satellite cells is altered (Mitchell et al., 2010). We observed that the increase in PICs is present at birth prior to any detectable loss of satellite cells indicating that a change in the PIC population is an early event following compromised satellite cell function in the *Pax7* mutant mouse (Mitchell et al., 2010). Furthermore, we have shown that PICs encompass the fibroadipogenic progenitors (FAPs) as well as a population of myogenically competent interstitial cells (myoPICs) (Pannérec et al., 2013). Since FAPs have already been shown to exert a promyogenic effect upon activated satellite cells but also underlie the fibrotic and adipogenic response in muscle tissue in pathological settings (Joe et al., 2010; Uezumi et al., 2010, 2011), we set out to determine whether these populations were altered in response to satellite cell depletion. Consistent with our previous observations, we observed that PICs and satellite cells are present in a ~1:1 ratio in normal resting muscle (Figure 1H). Following AcvR2B inhibition, this ratio does not display a significant change despite the increase in cell number suggesting a tight regulation of the stem cell niche in both normal and hypertrophic muscle. In contrast, depletion of the satellite cell compartment resulted in an alteration of the PIC/satellite cells ratio in favor of PICs (~7:1) however, we observed that RAP-031 treatment resulted in a PIC/satellite cells ratio of ~17:1 in satellite cell-depleted muscle (Figure 1H). Analyses performed on younger mice (5 week-old) (Supplementary Figure 2A) also showed an increase in both satellite cells and PICs following RAP-031 treatment (Supplementary Figure 2B), however PICs displayed a more pronounced increase as compared to satellite cells, leading to an altered ratio in favor of PICs (Supplementary Figure 2C). Moreover, we observed that the proportions of FAPs (PW1^pos^PDGFRα^pos^) and myoPICs (PW1^pos^PDGFRα^neg^) were unchanged in RAP-031 as compared to vehicle-injected mice (Supplementary Figures 2D–F), indicating that both PW1 expressing interstitial populations were equally increased after AcvR2B blockade. Taken together, these data indicate that AcvR2B pathway inhibition has a profound impact on the muscle stem cell niche in intact as well as in satellite cell-depleted muscles.

### AcvR2B inhibition restores the regenerative capacity and inhibits fibrosis/adipogenesis in muscle containing few satellite cells

**Figure 2:**
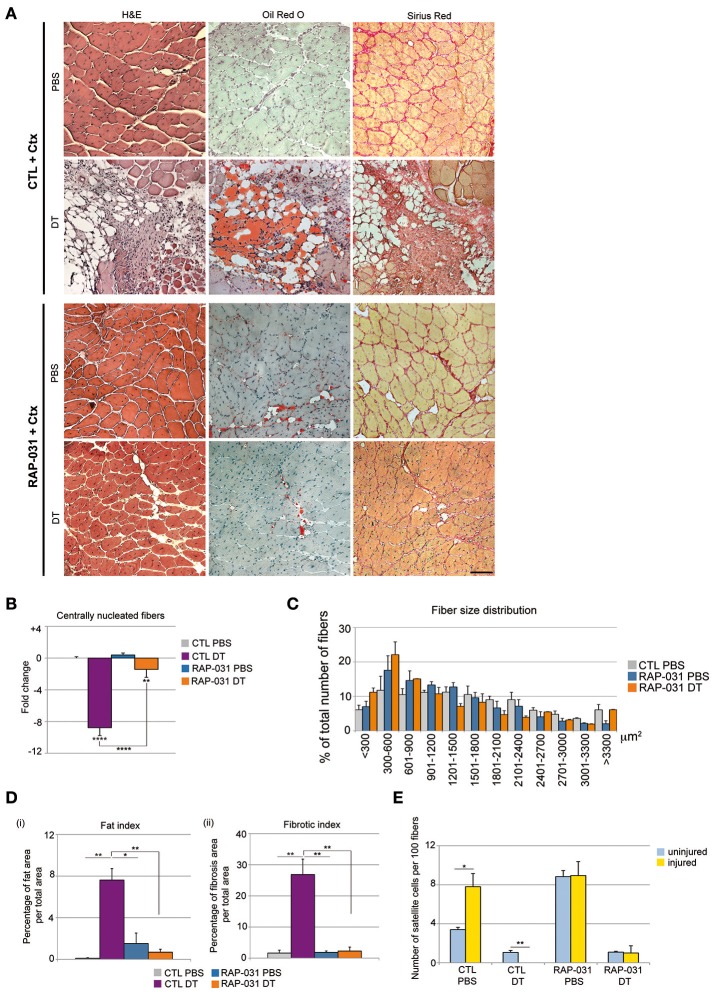
AcvR2B pathway inhibition improves overall fiber regeneration and ameliorates tissue homeostasis in injured satellite cell-depleted muscles. **(A)** Cross-sections of TA muscle from CTL (Upper) and RAP-031 (Lower) *Pax7*^*DTR*/+^ mice injected with DT or PBS stained with haematoxylin and eosin (Left), Oil-Red O (Middle) and Sirius Red (Right). Regeneration is more efficient in RAP-031 DT mice as compared to CTL DT mice, with a marked reduction in fat and fibrotic tissue deposition. Scale bar, 85 μm. **(B)** Quantitative analysis of regenerative capacity of TA muscles shown in **(A)**. The number of centrally nucleated fibers per field in TAs from CTL mice injected with PBS was considered as the baseline (zero) and values represent the fold change ± s.e.m. normalized accordingly. Muscle fiber regeneration is significantly rescued in RAP-031 DT mice as compared to CTL DT mice. Five randomly chosen fields from one mid-belly TA section were analyzed for each animal, *n* = 3 animals were analyzed for each group. **(C)** Fiber size distribution in CTL PBS (gray), RAP-031 PBS (blue), and RAP-031 DT (orange) TAs. Satellite cell-depleted muscles exposed to RAP-031 before injury exhibited regenerated fibers with a reduced size as compared to intact muscles. Values represent the mean number ± s.e.m. per 100 fibers for each size. *N* = 3 animals for each group. **(D)** Quantitative analysis of fat **(i)** and fibrotic **(ii)** areas of muscles showed in **(A)**. One mid-belly TA section was analyzed with ImageJ software for each animal and *n* = 3 animals were analyzed for each group. Fat areas were determined by Oil-Red O staining and fibrotic areas were determined by Sirius-Red staining. Values represent the percentage of fat **(i)** or fibrotic **(ii)** area on whole muscle area. **(E)** Quantification of satellite cells per 100 fibers in TA from uninjured (blue) and injured (yellow) *Pax7*^*DTR*/+^ CTL and RAP-031 mice injected with PBS or DT. Satellite cells were undetectable after injury in CTL DT muscle, however RAP-031 treatment was able to prevent satellite cell loss. Satellite cells were determined as Pax7^pos^ cells underneath the basal lamina. At least 5 randomly chosen fields, corresponding to at least 300 fibers, were counted for each animal, *n* = 3 animals were considered for each group. ^*^p < 0.05, ^**^p < 0.01, ^****^p < 0.001.

Satellite cell depletion results in a severely compromised regenerative response coupled with pronounced fibrosis and fat deposition (Lepper et al., 2011; Sambasivan et al., 2011) and this outcome is strikingly similar to late stage degenerative myopathies (Maier and Bornemann, 1999; Shefer et al., 2006; Tabebordbar et al., 2013). FAPs have been proposed to exert a promyogenic role on satellite cells during regeneration but also to directly contribute to fat and fibrosis in diseased muscle (Joe et al., 2010; Uezumi et al., 2010; Murphy et al., 2011), therefore we sought to determine whether the changes observed in the PICs population, which includes the FAPs, in both intact and satellite cell depleted muscles following RAP-031 treatment reflected a general change in the stem cell niche that in turn may provide a more favorable regenerative environment. Previously we showed that PICs and satellite cells have distinct transcriptomes (Pannérec et al., 2013). Closer examination of these previous data revealed a striking reciprocal expression of TGFβ ligands and their receptors in PICs and satellite cells although these earlier studies were performed on cells isolated from juvenile muscles and without separating the population based upon PDGFRα expression (Pannérec et al., 2013). Nonetheless, the close overlap between juvenile and adult satellite cell transcriptomes (Pannérec et al., 2013) prompted us to examine the expression of specific TGFβ family members and receptors in adult satellite cells, FAPs and myoPICs. Semi-quantitative PCR analyses revealed that adult satellite cells express activinA and MST (Supplementary Figure 3A), which are factors known to inhibit myogenesis and induce muscle atrophy (McPherron et al., 1997; Lee et al., 2010). In contrast, FAPs expressed FST and IGF-1 (Supplementary Figure 3A), which have been demonstrated to exert a promyogenic effect on muscle fibers and activated satellite cells (Glass, 2010; Lee et al., 2010) (Supplementary Figure 3A). We note that the myoPICs do not show this reciprocal expression of TGFβ family members, although they express different extracellular and intracellular players of the TGFβ pathway (Supplementary Figure 3A), suggesting a response to TGFβ pathway manipulation. Furthermore, we observed that satellite cell capacity to form clones *in vitro* is dramatically reduced when these cells are cultured in presence of increasing doses of exogenous MST (Supplementary Figures 3B,C), consistent with previous observations (McCroskery et al., 2003). We found that the decline of satellite cell clone-forming capacity in presence of MST can be reversed by the concomitant co-culture with PICs (Supplementary Figures 3B,C) and that this rescue can be abrogated by the addition of blocking antibodies against FST and IGF-1 to the medium (Supplementary Figure 3C). Taken together, these data are consistent with a model in which the PICs are a key cellular constituent of the satellite stem cell niche and serve to provide a promyogenic effect in healthy muscle in response to injury (Joe et al., 2010; Uezumi et al., 2010; Murphy et al., 2011; Mozzetta et al., 2013). We found here that PICs are strongly increased in number in satellite cell-depleted muscle following RAP-031 treatment as compared to CTL DT-injected mice (Figure 1G), while satellite cell number did not change and remained at a level of 30% of the intact muscle population (Figure 1F). Satellite cell-depleted TA muscles and control (PBS-injected) contralateral muscles were therefore injured by cardiotoxin (Ctx) 1 day before the last RAP-031 or vehicle injection and the level of regeneration was assessed at 2 weeks following injury. As expected, all RAP-031-treated (*n* = 9) and CTL (*n* = 9) TA muscles injected with PBS displayed robust myofiber regeneration while CTL satellite cell-depleted muscles displayed severely compromised regeneration coupled with fat deposition and fibrosis (Figure 2A). We observed rare and highly restricted regions of newly regenerated fibers in only 2 of the 5 CTL satellite cell depleted muscles (CTL DT) suggesting that restricted surviving satellite cells can be mobilized locally but fail to contribute significantly toward replacing muscle tissue. In contrast, RAP-031 treatment of satellite cell-depleted muscles resulted in a marked rescue in overall regeneration coupled with a near complete inhibition of fat formation and fibrosis in 7 out of 9 samples (Figure 2A). We note that depletion of satellite cells in vehicle-injected mice resulted in a strong decrease in the number of centrally nucleated fibers after injury compared to muscles injected with PBS (8.8-fold), whereas this decrease was less than 1.5-fold in RAP-031 DT mice as compared to CTL PBS muscles (Figure 2B). Despite the rescue in regenerative capacity following RAP-031 treatment, satellite cell-depleted muscles exhibited a 30% reduced fiber size as compared to normal intact muscles (median value of fiber area distribution: CTL PBS = 1,455.13 ± 175.68 μm^2^; RAP DT = 1003.80 ± 136.18 μm^2^) (Figure 2C), indicating that AcvR2B pathway inhibition via RAP-031 administration before injury is able to rescue overall regeneration capacity in satellite cell-depleted muscles, but does not rescue satellite cell depletion-induced muscle mass loss after injury. We note that while RAP-031 treatment is able to increase fiber size in homeostatic intact muscles (Figure 1D), it does not induce muscle fiber hypertrophy after injury (median value of fiber area distribution: CTL PBS = 1,455.13 ± 175.68 μm^2^; RAP PBS = 1,208.55 ± 190.74 μm^2^) (Figure 2C) suggesting that the AcvR2B signaling pathway in resting and regenerating muscle acts differently. Moreover, in RAP-031 satellite cell-depleted muscle, regeneration was coupled with a marked decrease of fat as well as fibrotic zones to levels comparable to CTL intact muscle (Figure 2D).

We next compared the number of satellite cells in injured and uninjured muscles. As expected (Wang and Rudnicki, 2012; Yin et al., 2013), satellite cells were increased in CTL PBS-injected muscle after injury (Figure 2E). In contrast, satellite cell numbers were below the level of detection in CTL DT-injected contralateral muscles after injury (Figure 2E), indicating that the failure of regeneration was accompanied by a complete loss of satellite cell self-renewal after Ctx injection. We found that RAP-031 treatment did not significantly change the amount of satellite cells present in the regenerated intact muscle (Figure 2E) indicating that regeneration of RAP-031 treated muscle is accompanied by the re-establishment of the normal intact satellite cell population number. Similarly, satellite cell-depleted muscles pretreated with RAP-031 gave rise to new muscle with a satellite cell population that was restored to ~30% of the intact muscle population (Figure 2E). These data demonstrate that inhibition of the AcvR2B pathway in muscle tissue containing a reduced population of satellite cells is able to rescue regeneration but that the satellite cell population is only restored to pre-injured levels.

### AcvR2B inhibition rescues residual satellite cell regenerative capacity

**Figure 3:**
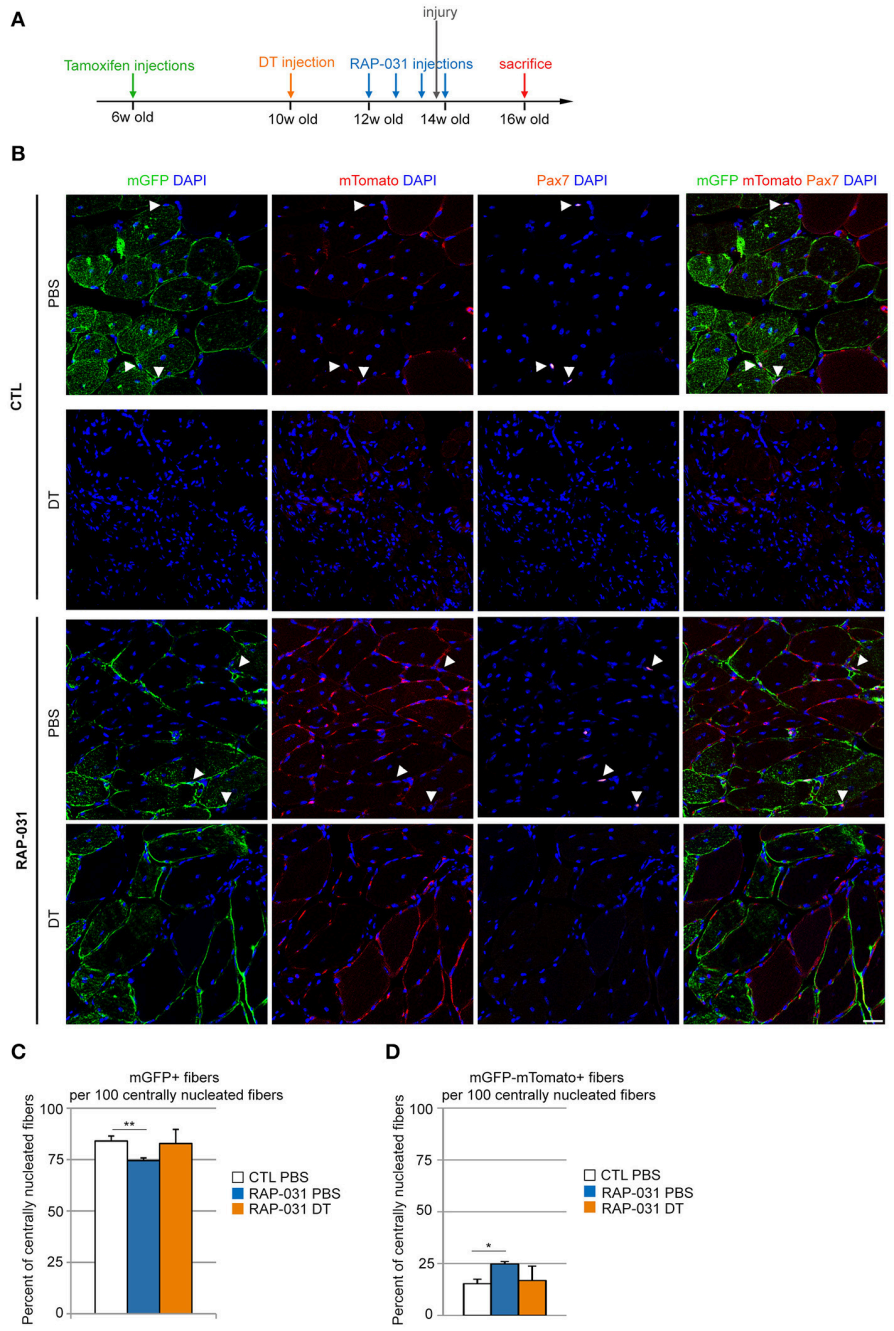
Regeneration in satellite cell-depleted muscles following AcvR2B pathway inhibition is mainly executed by the residual satellite cells. **(A)** Strategy: 6 week-old Pax7^DTR/+^:Pax7CreER^T2^:ROSA^mTmG^ mice were injected with tamoxifen to label satellite cells. 4 weeks later, the right TA was injected with DT to deplete the satellite cell pool. The contralateral muscle was injected with PBS. After 2 weeks, mice were injected with either RAP-031 or vehicle (CTL) twice a week for 2 weeks. The day before the last injection of RAP-031 or vehicle both TAs were injured by cardiotoxin injection. Mice were sacrificed 2 weeks after injury. **(B)** Representative images of cross-sections from injured TA muscles of CTL (Upper) and RAP-031 (Lower) mice injected with DT or PBS as described in **(A)**, immunostained for mGFP (green) to identify satellite cell-derived fibers and Pax7 (orange) to identify satellite cells. mTomato fluorescence is shown in red, DAPI staining (blue) identifies nuclei. The majority of regenerating fibers are satellite cell-derived (green) regardless of treatment. White arrowheads: satellite cells. Scale bar, 30 μm. **(C,D)** Quantification of the number of mGFP^pos^ **(C)** and mGFP^neg^mTomato^pos^ **(D)** fibers per 100 centrally nucleated fibers for CTL and RAP-03 mice injected with PBS or DT, as described in **(A)**. Values represent the mean number of positive fibers ± s.e.m. per 100 centrally nucleated fibers. Random images were captured at 20X magnification and at least 5 different fields for each section were counted, *n* = 3 animals were considered for each group. ^*^p < 0.05, ^**^p < 0.01.

Our results raised the question as to whether the regeneration we observed in RAP-031 treated satellite cell-depleted muscle was primarily due to the activation of the residual satellite cells or due to the recruitment of non-satellite cell myogenic populations. We demonstrated previously that PICs contain a population of myogenic cells and several studies have shown the presence of myogenic cells other than satellite cells in normal muscle tissue however their participation in regeneration is unproven (Mitchell et al., 2010; Pannérec et al., 2013). To determine the origin of regenerating myofibers, we crossed *Pax7*^*DTR*/+^ mice with *Tg:Pax7CreER*^*T*2^ mice (Mourikis et al., 2012) and with reporter line *ROSA26*^*mTomato*/*mGFP*^ (ROSA^mTmG^) (Muzumdar et al., 2007). The resulting Pax7^DTR/+^:Pax7CreER^T2^:ROSA^mTmG^ mice express a tamoxifen-inducible Cre (Metzger and Chambon, 2001) such that *Pax7*-expressing satellite cells and their progeny are specifically marked by membranous GFP (mGFP), while all the other cells are marked by membranous Tomato (mTomato) (Mourikis et al., 2012). To verify the efficiency of Cre-mediated labeling of the satellite cell population, 6 week-old Pax7CreER^T2^:ROSA^mTmG^ mice were injected with tamoxifen and sacrificed 6 weeks later (Supplementary Figure 4A). We stained TA muscles for mGFP and Pax7 and observed that all Pax7-positive cells were positive for mGFP (Supplementary Figure 4B) indicating that the expression of mGFP labeled the entirety of the Pax7^pos^ satellite cell population. In addition, we observed mGFP^pos^ sublaminal monuclear cells that did not express Pax7 at detectable levels indicating that mGFP labeling identified a larger set of satellite cells, which displayed low or undetectable levels of Pax7 at the time of the analysis (Supplementary Figure 4B). Taken together, we conclude that nearly 100% of the satellite cell population was marked by mGFP expression such that the Pax7^DTR/+^:Pax7CreER^T2^:ROSA^mTmG^ mice provides a genetic tool for determining whether newly regenerated fibers are derived from satellite cells in which case fibers are predicted to express mGFP whereas the presence of mGFP^neg^mTomato^pos^ regenerating fibers would indicate a contribution of non-satellite progenitor cells. We then used 6 week-old Pax7^DTR/+^:Pax7CreER^T2^:ROSA^mTmG^ mice injected with tamoxifen to activate mGFP expression in satellite cells followed by a single injection of DT in the right TA and PBS in the contralateral muscle 4 weeks after the last tamoxifen injection. Muscles were allowed to recover for 2 weeks and then treated with RAP-031. Muscles were injured by cardiotoxin 1 day before the last RAP-031 injection and the regeneration was assessed 2 weeks later (Figure 3A). In satellite cell-depleted muscles from RAP-031 mice, we observed that the majority of centrally nucleated fibers (>80%) were mGFP^pos^ (Figures 3B,C), comparable to vehicle (CTL) PBS and RAP-031 PBS conditions (Figures 3B,C), indicating that the reduced pool of satellite cells can be mobilized by RAP-031 treatment in satellite cell depleted muscles after injury. While these data confirm previous studies demonstrating that the satellite cells are the major muscle progenitor (Relaix and Zammit, 2012) we note that ~15% of the centrally-nucleated fibers in intact muscle are exclusively marked by mTomato (Figure 3D), strongly suggesting a contribution of non-satellite cells. Interestingly, we observed a 10% increase in the number of mGFP^neg^mTomato^pos^ centrally nucleated fibers and a concomitant 10% decrease of mGFP^pos^ centrally nucleated fibers in intact muscles from RAP-031 treated mice as compared to CTL mice (Figures 3C,D), suggesting that AcvR2B pathway inhibition stimulates non-satellite cell recruitment. Alternatively, there may be a small population of satellite cells that are resistant to Cre labeling that accounts for the generation of tomato positive fibers.

## Discussion

In addition to playing a key role during myofiber regeneration, satellite cells can fuse to existing myofibers during muscle hypertrophy leading to a stabilization of the larger myofiber mass (Amthor et al., 2009; McCarthy et al., 2011; Lee et al., 2012; Wang and McPherron, 2012). In this study, we found that a ~70–80% reduction in the number of satellite cells triggers a decrease in myofiber size suggesting a role for satellite cells in maintaining fiber size. Interestingly, previous studies showed that depletion of satellite cells in adult mice (4-month old) did not reduce muscle fiber size nor exacerbate age-related sarcopenia (Fry et al., 2015; Murach et al., 2017), however depletion performed at 2.5 months of age (this study) resulted in fiber size reduction. Moreover, we found that myofiber hypertrophy is limited in muscles containing fewer satellite cells following AcvR2B pathway inhibition. Our observations are therefore consistent with a previous study in which satellite cell depletion in young mice (2.5 months old) abrogates subsequent mechanical overload-induced fiber hypertrophy (Murach et al., 2017), while satellite cells are usually reported to be dispensable in hypertrophy processes at later ages (>4 months) (Murach et al., 2017, 2018). Taken together, these data suggest an active, age-dependent role for satellite cells in normal late postnatal muscle growth.

The central question of this study was why a reduced satellite cell population cannot properly regenerate and our observations here suggest that the microenvironment is altered in response to a decline in satellite cells. Previous reports demonstrated that as few as 10 satellite cells can generate hundreds of myofibers when engrafted in healthy muscle. It has also been proposed that the diseased muscle environment does not favor optimal satellite cell function (Mann et al., 2011; Serrano et al., 2011; Pannérec et al., 2012; Tabebordbar et al., 2013; Yin et al., 2013). Consistent with this proposal, it has been shown that regenerative capacity declines sharply when the satellite cell pool declines to 50% of initial satellite cell complement in the double mouse mutant for utrophin and dystrophin (Lu et al., 2014). Our results confirm satellite cells contribute to muscle homeostasis.

The importance of niche cells has been demonstrated in multiple tissues which serve as cellular partners to govern progenitor activation, self-renewal and survival (Leatherman, 2013). We demonstrated previously that PW1/Peg3 expression can be used to identify a large array of adult progenitor/stem cells including associated niche cells (Besson et al., 2011). As one example, PW1/Peg3 is expressed in the hair follicle bulge cells that are a major reservoir of skin progenitors as well as in closely associated dermal papilla cells (Besson et al., 2011, 2017) which have a dual role as mesenchymal stem cells and as critical niche support cells (Driskell et al., 2011; Wong et al., 2012). We suggest that the expression of PW1/Peg3 in the satellite cells and a subset of interstitial cells including most notably the FAPs, defines a similar niche in skeletal muscle.

While our lineage tracing analyses confirms that satellite cells are the major source of regenerated muscle in all cases, several non-satellite cell populations have been shown to have pronounced myogenic activity (Pannérec et al., 2012). In intact skeletal muscle, ~15% of the regenerated muscle fibers do not express the satellite cell lineage marker following damage and increase to ~25% following AcvR2B pathway inhibition. Since all Pax7-positive cells express GFP following tamoxifen injection, our data suggest that non-satellite cell progenitors participate directly in muscle regeneration but require an intact satellite cell compartment although lineage-specific markers for the myoPIC population is required to assess the origin of these cells.

**Figure 4:**
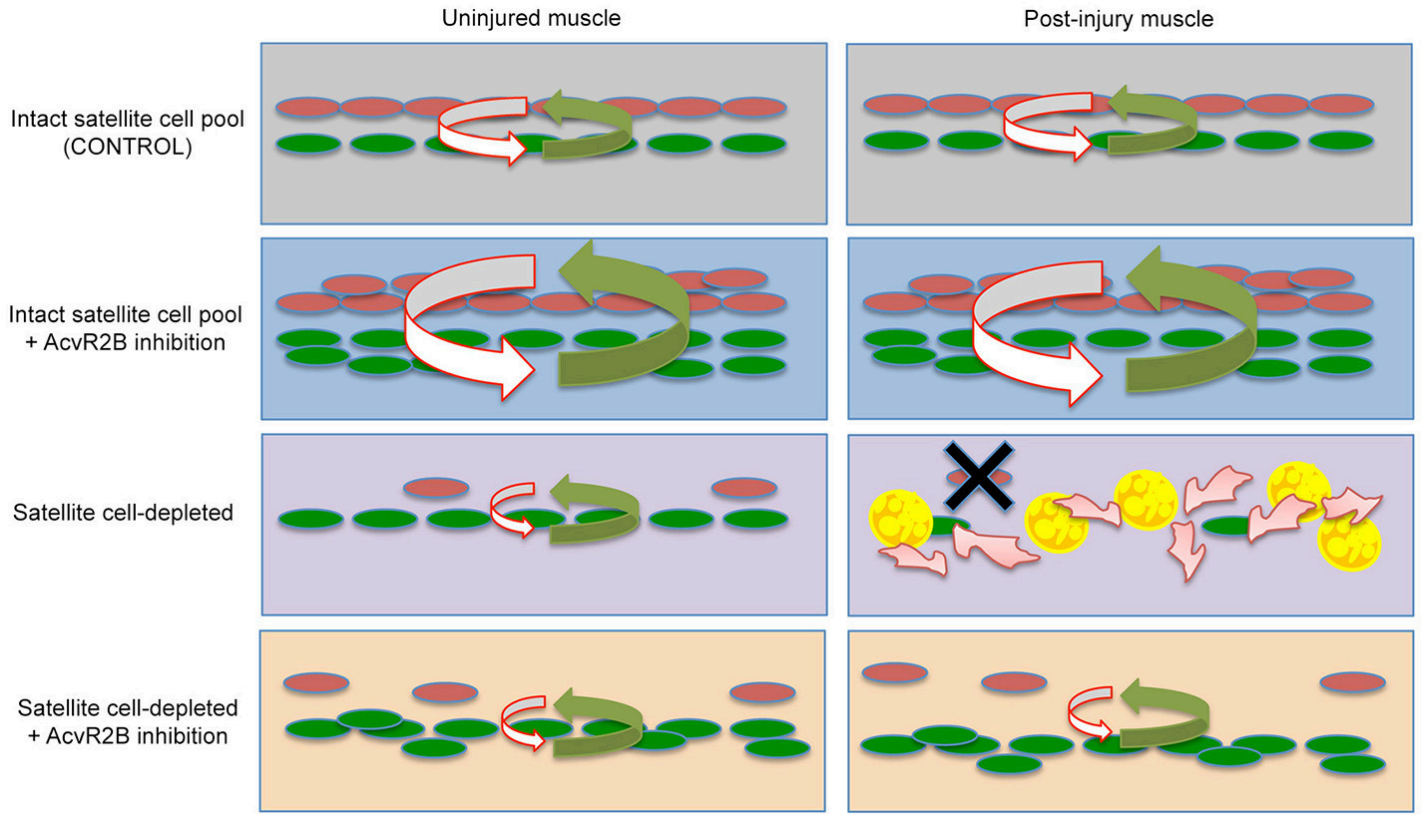
Proposed cellular model of satellite cell and stem cell niche interactions. Top Row- In resting muscle containing an intact population of satellite cells **(Left)**, pro-myogenic signals (green arrow) from PICs/FAPs (green), and anti-adipogenic signals (red arrow) from satellite cells (red) are kept in a tight balance, which is maintained after injury **(Right)**. Second Row- Following AcvR2B inhibition, PICs and satellite cell populations expand, however pro- and anti-myogenic balance in maintained before and after injury. Third Row- Satellite cell depletion disrupts the balance and overall levels of factors regulating the niche **(Left)** and signaling within the niche is completely deregulated following injury **(Right)**. This results in a complete loss of satellite cells and consequently a loss of satellite cell derived anti-adipogenic signals allowing FAP progression into fat and fibrosis **(Right)**. Fourth Row- AcvR2B inhibitor treatment of satellite cell depleted muscle reinforces the niche **(Left)** and allows for satellite cell myogenic progression/self-renewal and consequently the maintenance of a minimal balance of factors required to restore the muscle tissue and stem cell niche **(Right)**.

It has been described previously that satellite cells inhibit adipogenic differentiation of FAPs and that FAPs are promyogenic for satellite cells (Joe et al., 2010; Uezumi et al., 2010, 2011). We report here that in normal resting muscle, satellite cells express myogenic inhibitory factors such as MST and activinA, whereas FAPs express promyogenic factors including FST and IGF-1. These observations provide a framework (Figure 4) in which satellite cells are present in a niche which includes the FAPs and that the balance of pro- and anti-myogenic signals keeps myogenesis and fibro/adipogenesis tightly regulated.

The AcvR2B pathway has been a focus for the development of therapeutics for degenerative muscle diseases due to the recognition that these molecules antagonize myostatin and activin activity (Glass, 2010; Zhou and Lu, 2010; Ceco and McNally, 2013). A recent study has revealed that histone deacetylase (HDAC) inhibitors provide a promising route to treat muscular dystrophy in murine models via the induction of follistatin in interstitial muscle cells consistent with our results here showing that muscle mass is regulated by complex interactions between multiple cells types (Mozzetta et al., 2013). It is of interest that the beneficial effects of HDAC inhibitors decrease efficacy with age (Mozzetta et al., 2013). Similarly, we report here that AcvR2B inhibition in young mice (5 weeks) causes an increase in the PIC/satellite cell ratio disrupting the typical 1:1 ratio, while a 1:1 ratio is maintained in 10-week-old mice. Taken together, these data indicate that changes in regenerative capacity and stem cell competence during postnatal life reflect a loss of plasticity in the stem cell niche.

## Conclusions

Our results point to a mechanistic convergence for satellite cells governing both regeneration and the maintenance of muscle fiber size. Whereas 20–30% of the normal population of satellite cells is insufficient to give rise to new muscle following damage, this reduced population can execute the regeneration program if the AcvR2B pathway is pharmacologically targeted. We propose that pharmacological targeting of the AcvR2B pathway restores a balance of regulatory factors within the muscle stem cell niche, in part through the regulation of cells that exert a promyogenic effect as well as cells capable of generating fibrotic and fat tissue, such as PICs/FAPs, that can interfere with the proper reassembly of muscle tissue (Figure 4), rather than directly promoting regeneration through the activation of satellite cells. This idea is also supported by our observation that AcvR2B inhibition treatment before injury does not alter the activation status of either the satellite cell population nor other cells types (such as PICs/FAPs) suggesting that all the cellular components of the muscle stem cell niche are important for proper regeneration. The capacity to restore the regenerative potential of muscle containing a reduced progenitor population through pharmacological intervention will impact the design of future therapeutic approaches for degenerative myopathies.

## Author contributions

LF, AP, RC, DO, and BG-M performed experiments with reagents provided by JL and JS and expertise in using the mouse models was provided by VB, BG-M, and ST. All authors designed experiments, analyzed and interpreted data. LF, AP, RC, GM, and DS prepared the manuscript.

### Conflict of interest statement

The authors declare that the research was conducted in the absence of any commercial or financial relationships that could be construed as a potential conflict of interest.

## Supplementary material

Supplementary Figure 1 Representative pictures of immuno-stainings in uninjured TA muscles. Representative images of cross-sections from uninjured TA muscles of CTL **(Upper)** and RAP-031 **(Lower)** mice injected with DT or PBS, immunostained for PW1 (green), M-cadherin (red), Laminin (orange). DAPI staining (blue) identifies nuclei. Satellite cells (white arrowheads) are identified as M-cadherin^pos^ cells. PICs (yellow arrowheads) are identified as PW1^pos^ interstitial cells. Scale bar, 20 μm.

Supplementary Figure 2 RAP-031 treatment of intact muscle from young mice alters the muscle stem cell niche. **(A)** Strategy: 5 week-old male wild-type mice were injected intraperitoneally with RAP-031 or vehicle (CTL) every 3 days and sacrificed 2 weeks after the first injection. **(B)** Quantification of satellite cells and PICs per 100 fibers in CTL and RAP-031 TA muscles showed an increase of both cells types after RAP-031 treatment. **(C)** Quantification of progenitor cells shown in **(B)** for CTL and RAP-031 *Tibialis Anterior* revealed an altered ratio between PICs (green) and satellite cells (red) in RAP-031 vs. CTL. For **(B,C)** PICs were determined as interstitial PW1^pos^Pax7^neg^ cells, satellite cells as Pax7^pos^ cells underneath the basal lamina. **(D,E)** Flow cytometric analyses of single cells from 7-week old limb muscles from CTL **(D)** or RAP-031-injected **(E)** PW1^nLacZ^ mice following the protocol described in **(A)**. Cells were stained for CD45, Ter119, CD34, Sca1, PDGFRα, and C_12_FDG (to reveal PW1 expression via β-galactosidase activity), as reported in Pannérec et al. (2013). CD45^neg^Ter119^neg^ were selected. The gates used to isolate FAPs (CD34^pos^Sca1^pos^PW1^pos^PDGFRα^pos^) and myoPICs (CD34^pos^Sca1^pos^PW1^pos^PDGFRα^neg^) are shown. Satellite cells (SAT) are also shown in red (CD34^pos^Sca1^neg^). **(F)** Number of FAPs (PW1^pos^PDGFRα^pos^) and myoPICs (PW1^pos^PDGFRα^pos^) from CTL and RAP-031 mice as presented as the mean percentage ± s.e.m. per 100 PICs (CD34^pos^Sca1^pos^PW1^pos^) cells from at least 3 independent experiments. ^*^*p* < 0.05, ^**^*p* < 0.01, and ^***^*p* < 0.001.

Supplementary Figure 3 Adult PICs and satellite cells express TGFβ pathway related genes. **(A)** Semi-quantitative PCR of selected genes involved in the TGFβ and IGF-1 pathway in freshly sorted adult satellite cells (SAT), FAPs (PW1^pos^PDGFRα^pos^), and myoPICs (PW1^pos^PDGFRα^neg^) sorted as shown in (Supplementary Figure 1D). PC, Positive Control: whole muscle extract from 7 week-old PW1nLacZ mice, except for activin α subunit, IGF-1 IEa and IEb (whole liver extract from 7 week-old PW1nLacZ mice) and for TGFβR2 (whole brain extract from 7 week-old PW1nLacZ mice). **(B)** Schematic transwell membrane system used: PICs (green) were plated in the upper well on a semipermeable membrane (insert) and satellite cells (red) were plated in the lower chamber. **(C)** Quantitative analyses of satellite cell proliferation in growth medium containing 0, 20, 200 ng/ml, or 2 μg/ml of recombinant myostatin. Satellite cells were cultured alone (red bars) or in the presence of PICs and isotype-matched IgG (gray bars). Satellite cells and PICs were co-cultured in presence of a blocking antibody to follistatin (αFST, blue bars) or to IGF-1 (αIGF-1, black bars) or blocking antibodies to IGF-1 and FST together (αFST + aIGF1, white bars). Large colonies (>12 cells) were counted and shown as a percentage of the number of total colonies.

Supplementary Figure 4 Efficiency of inducible *Pax7*-driven Cre mediated recombination of Rosa^mTomatomGFP^ locus. **(A)** 6 week-old Pax7CreER^T2^:ROSA^mTmG^ mice were injected with tamoxifen every day for 4 days and sacrificed 6 weeks later. **(B)** Quantification analysis of satellite cells in mice treated as in **(A)**. **(i)** 100% of Pax7^pos^ satellite cells express mGFP. Values represent the mean number of GFP^pos^ and GFP^neg^ cells ± s.e.m. per 100 Pax7^pos^ cells. **(ii)** The majority (>80%) of GFP^pos^ mononuclear cells co-express Pax7, however <20% of the GFP^pos^ mononuclear cells are not co-labeling with Pax7. Values represent the mean number of Pax7^pos^ and Pax7^neg^ cells ± s.e.m. per 100 GFP^pos^ mononuclear cells. For quantification, immunostaining for Pax7 and GFP was performed on TA muscle section from *n* = 4 mice and at least 5 different randomly chosen fields were counted for each section, corresponding to an average number of fibers of >120 per section.

Supplementary Table 1 List of primers used for semi-qPCR.
