# Supplementary figures and images for: Inhibition of the Activin Receptor Type-2B Pathway Restores Regenerative Capacity in Satellite Cell-Depleted Skeletal Muscle
Source: Front Physiol. 2018 May 24;9:515. doi: 10.3389/fphys.2018.00515 (PMC5978452; doi:10.3389/fphys.2018.00515)

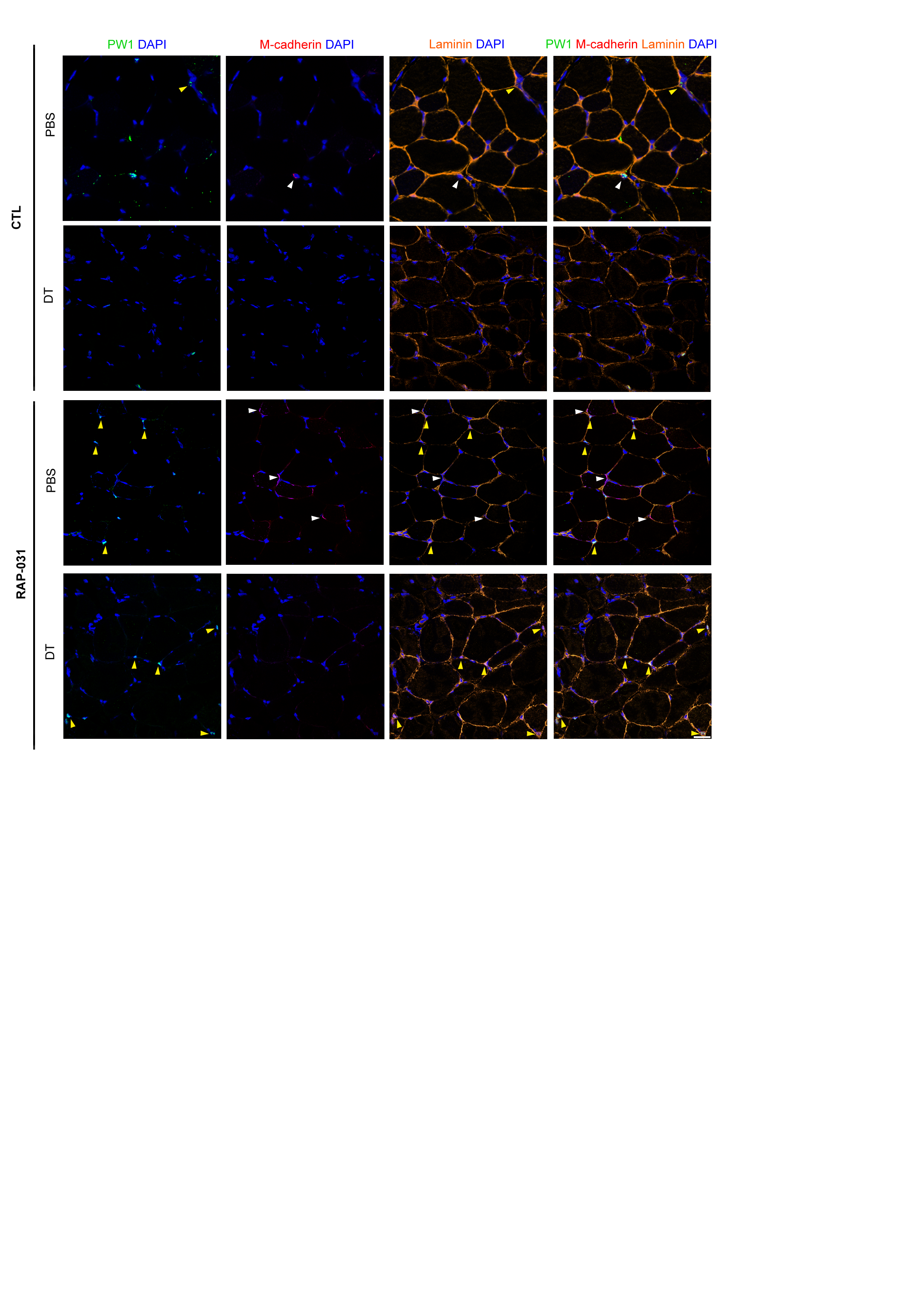

Supplement: Supplementary Figure 1 — Representative pictures of immuno-stainings in uninjured TA muscles. Representative images of cross-sections from uninjured TA muscles of CTL (Upper) and RAP-031 (Lower) mice injected with DT or PBS, immunostained for PW1 (green), M-cadherin (red), Laminin (orange). DAPI staining (blue) identifies nuclei. Satellite cells (white arrowheads) are identified as M-cadherinpos cells. PICs (yellow arrowheads) are identified as PW1pos interstitial cells. Scale bar, 20 μm. [file Image_1.TIF]
