# Supplementary material for: Inhibition of the Activin Receptor Type-2B Pathway Restores Regenerative Capacity in Satellite Cell-Depleted Skeletal Muscle
Source: Front Physiol. 2018 May 24;9:515. doi: 10.3389/fphys.2018.00515 (PMC5978452; doi:10.3389/fphys.2018.00515)
Supplement: Supplementary Table 1 — List of primers used for semi-qPCR. [file Table_1.docx]

**Supplementary Table 1**. List of primers used for semi-qPCR.

| **Gene** | **Forward primer** | **Reverse primer** |
| --- | --- | --- |
| 18S | CGGCTACCACATCCAAGGAA | TATACGCTATTGGAGCTGGAA |
| Myostatin | GGCTCAAACAGCCTGAATCCAA | CCAGTCCCATCCAAAGGCTTCAAA |
| Follistatin | CCCCAACTGCATCCCTTGTAAA | TCCAGGTGATGTTGGAACAGTC |
| Activin α subunit | ATGTATTCCGGCCATCCCAA | CTACCATGGCAGTAGTGGAA |
| Activin βA subunit | GAGAGGAGTGAACTGTTGCT | TACAGCATGGACATGGGTCT |
| Activin βB subunit | AGCCTCTGTTGCAGGCAA | TTCACGGGCCCAGGGTTCA |
| TGFβ1 | GCTGCGCTTGCAGAGATTAAA | TTGCTGTACTGTGTGTCCAG |
| TGFβ2 | CCAAAGACTTAACATCTCCCACC | GTTCGATCTTGGGCGTATTTC |
| TGFβ3 | GCTCTTCCAGATACTTCGAC | AGCAGTTCTCCTCCAGGTTG |
| ALK-2/AcvR1 | ATGACTACCTTCAGCTCACT | CTTCGCCAGAGAAGTTAATG |
| ALK-3/BMPR1A | TACACTGCCCCCTGTTGTTA | CTCTGGTGTCTAGTGTGGCA |
| ALK-4/AcvR1B | ACCGCTACACAGTGACCATT | TCTTCACATCTTCCTGCACG |
| ALK-5/TGFβR1 | ATCCATCACTAGATCGCCCT | CGATGGATCAGAAGGTACAAGA |
| AcvR2A | CTTAAGGCTAATGTGGTCTC | GACTAGATTCTTTGGGAGGA |
| AcvR2B | ATCGTCATCGGAAACCTCCC | CAGCCAGTGATCCTTAATC |
| TGFβR2 | CGTGTGGAGGAAGAACAACA | TCTCAAACTGCTCTGAGGTG |
| BMPR2 | AGATCTATCCTCTCCCTAAG | TTAGAATGGACTGCCCTGTC |
| Smad1 | ACTGAAGCCTCTGGAATGCT | GCGGTTCTTATTGTTGGACG |
| Smad2 | CGGAACCTGCATTCTGGTGTTCAA | CTCAGCAAACACTTCCCCACCTAT |
| Smad3 | ATTTTCGTCCAGTCTCCCAACTGC | GCCTTTGACGAAGCTCATACGGAT |
| Smad4 | AGGCAAAGGAGTGCAGTTGGAATG | TGACACTGCCGCAGATCAAAGA |
| Smad5 | ATGCCCAGCATATCCAGCAG | CAGAAGAAATGGGGTTCAGC |
| BMP-2 | TGCTCAGCATGTTTGGCCTGAA | AAACTCGTCACTGGGGACAGAACT |
| IGF-1 IEa (416 bp) & IEb (468 bp) | ATTTAAGATCTGCCTCTGTGACTTCTT | TCTTGTTTCCTGCACTTCCTCTACT |
| IGF-1 Eb (I/II) | CCCGTCCCTATCGACAAACAAGAA | ATCCTGCGGTGATGTGGCATT |
| IGF-2 | TGTCTTCATCCTCTTCCAGCCCCA | GCCAAAGAGATGAGAAGCACCAACA |
| IGF-1R | GAGAAAAGGGAATTTCGTCCCAAATAAAAGG | CTATGGTGGAGAGGTAACAGAGGTC |

Table 1
